# Supplementary figures and images for: Genome-Wide Identification and Analysis of TCP Gene Family among Three Dendrobium Species
Source: Plants (Basel). 2023 Sep 7;12(18):3201. doi: 10.3390/plants12183201 (PMC10538224; doi:10.3390/plants12183201)

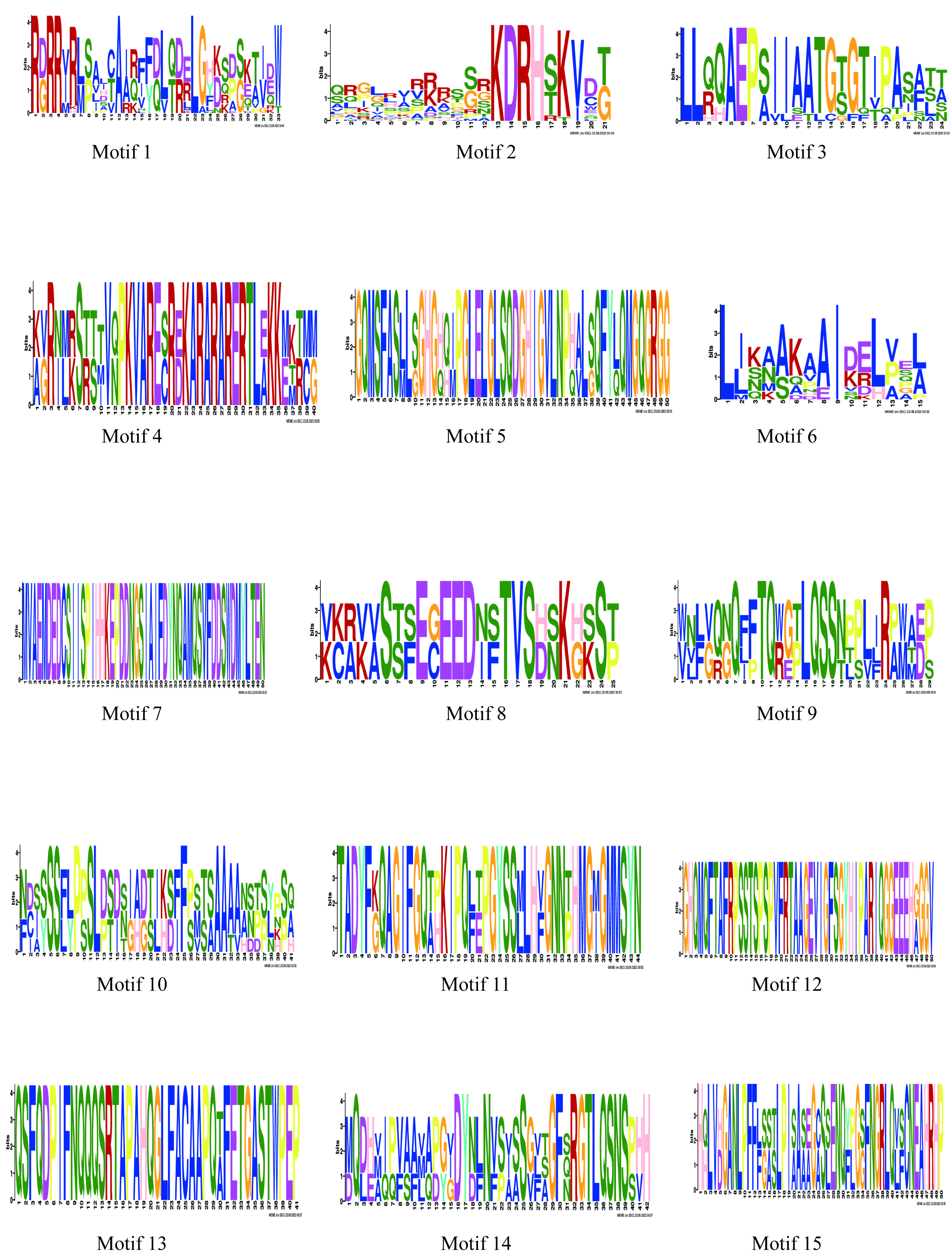

Supplement: Supplementary file 1 [file plants-12-03201-s001.zip › supplementary/Supplementary fig 1.tif]

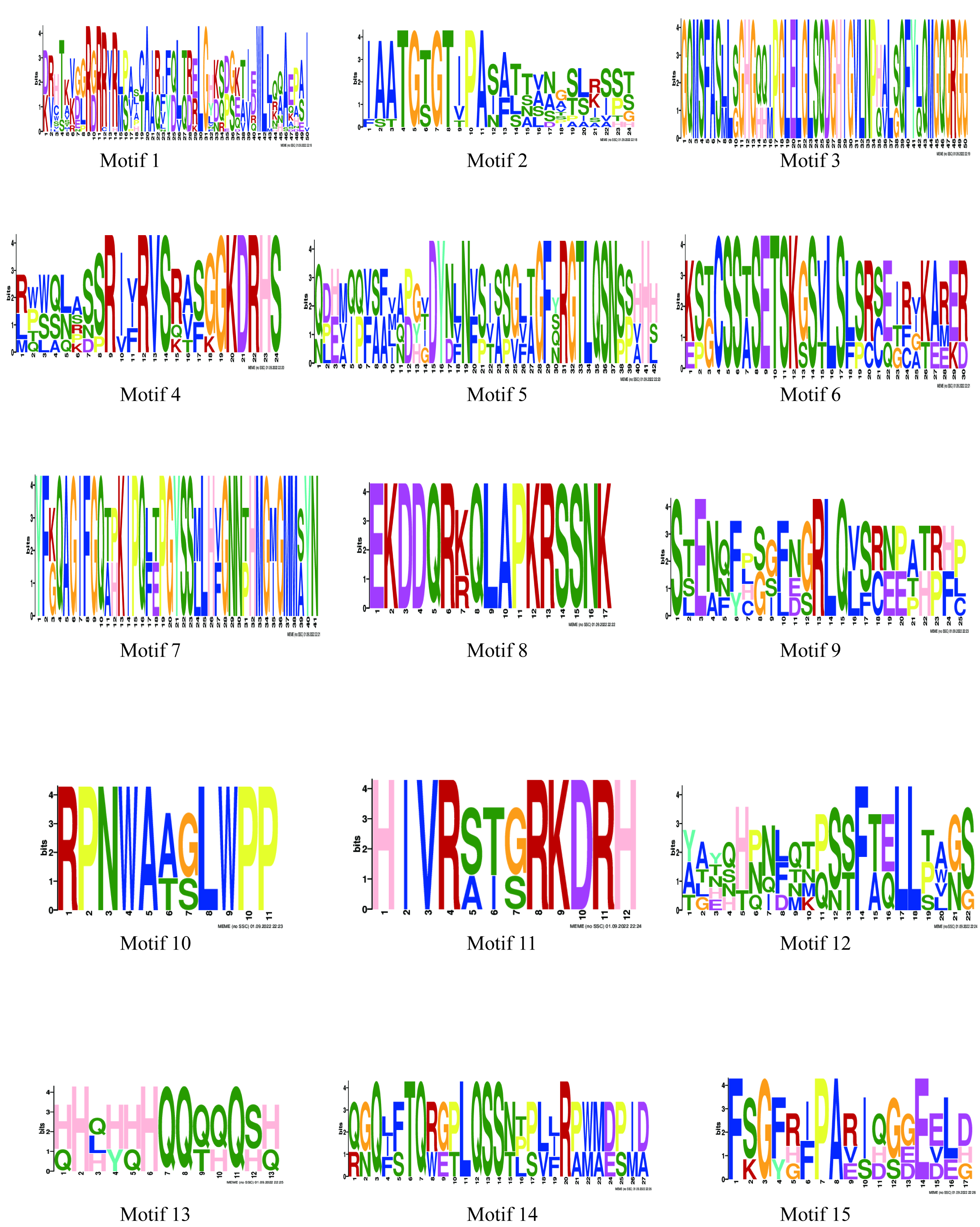

Supplement: Supplementary file 1 [file plants-12-03201-s001.zip › supplementary/Supplementary fig 2.tif]

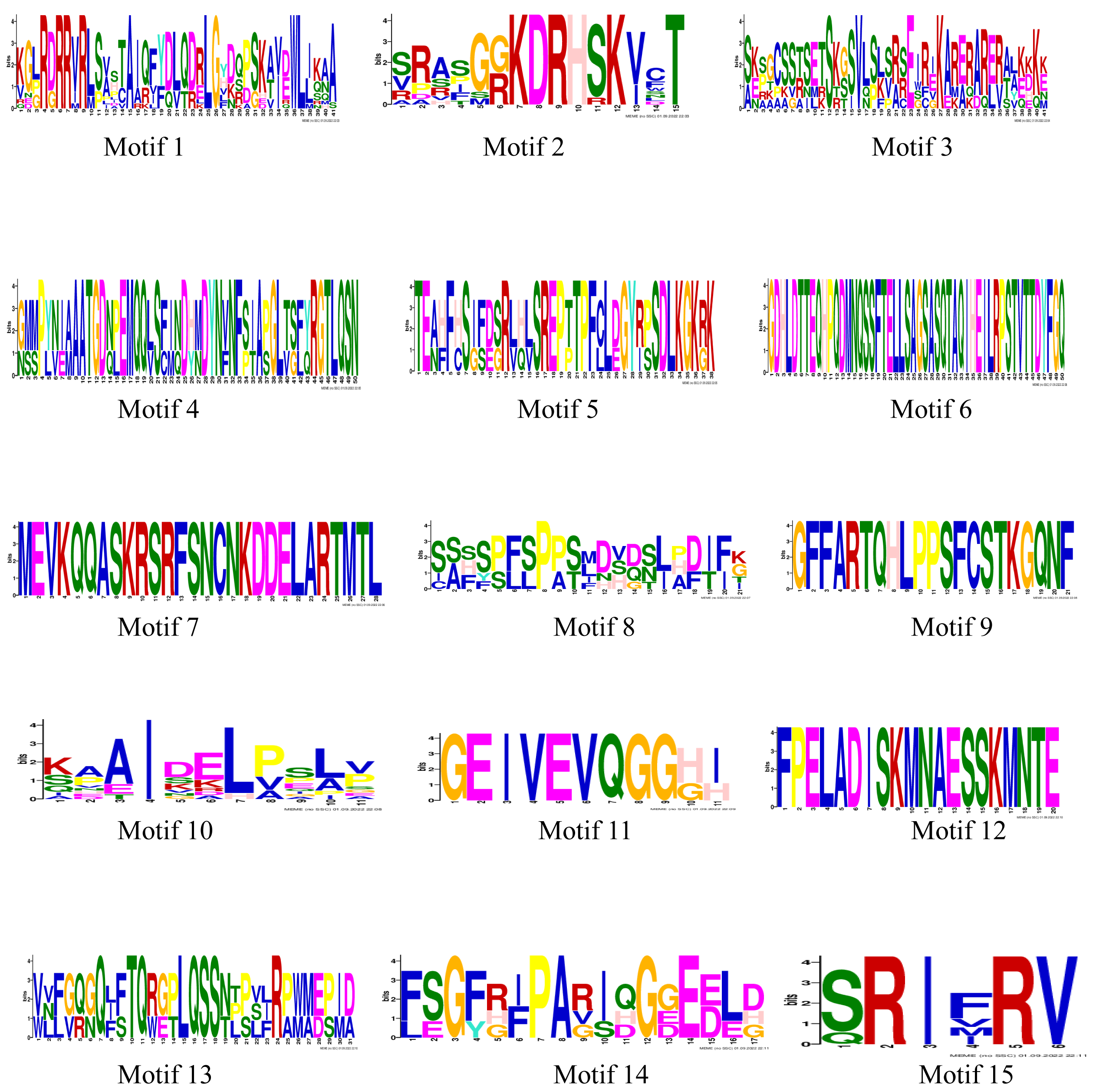

Supplement: Supplementary file 1 [file plants-12-03201-s001.zip › supplementary/Supplementary fig 3.tif]
